# Phagocytosis of Astaxanthin-Loaded Microparticles Modulates TGF*β* Production and Intracellular ROS Levels in J774A.1 Macrophages

**DOI:** 10.3390/md19030163

**Published:** 2021-03-19

**Authors:** Eleonora Binatti, Gianni Zoccatelli, Francesca Zanoni, Giulia Donà, Federica Mainente, Roberto Chignola

**Affiliations:** 1 Department of Biotechnology, University of Verona, Strada Le Grazie 15-Cv1, I-37134 Verona, Italy; eleonora.binatti@univr.it (E.B.); gianni.zoccatelli@univr.it (G.Z.); federica.mainente@univr.it (F.M.); 2 Sphera Encapsulation S.r.l., Strada Le Grazie 15-Cv1, I-37134 Verona, Italy; zanoni@spheraencapsulation.com (F.Z.); lab@spheraencapsulation.com (G.D.)

**Keywords:** astaxanthin, protein microparticles, macrophage targeting, oxidative stress, TGF*β*, inflammation, radiotherapy

## Abstract

Radiation-induced fibrosis is a serious long-lasting side effect of radiation therapy. Central to this condition is the role of macrophages that, activated by radiation-induced reactive oxygen species and tissue cell damage, produce pro-inflammatory cytokines, such as transforming growth factor beta (TGFβ). This, in turn, recruits fibroblasts at the site of the lesion that initiates fibrosis. We investigated whether astaxanthin, an antioxidant molecule extracted from marine and freshwater organisms, could help control macrophage activation. To this purpose, we encapsulated food-grade astaxanthin from *Haematococcus pluvialis* into micrometer-sized whey protein particles to specifically target macrophages that can uptake material within this size range by phagocytosis. The data show that astaxanthin-loaded microparticles are resistant to radiation, are well-tolerated by J774A.1 macrophages, induce in these cells a significant reduction of intracellular reactive oxygen species and inhibit the release of active TGFβ as evaluated in a bioassay with transformed MFB-F11 fibroblasts. Micro-encapsulation of bioactive molecules is a promising strategy to specifically target phagocytic cells and modulate their own functions.

## 1. Introduction

Astaxanthin (3,3${}^{\prime }$-dihydroxy-β-β${}^{\prime }$-carotene-4,4${}^{\prime }$-dione; ASX) is a xantophyll ketocarotenoid present in several marine and freshwater organisms, including microorganisms, crustaceans and fishes [1]. First discovered in lobsters, it was initially employed as feed in aquaculture since ASX confers the typical red-orange colour to salmonids [1]. Later, however, thanks to the discovery of its antioxidant activity, it was approved as a food supplement to promote human health [1]. In fact, as an antioxidant, ASX is 10 times more potent than other carotenoids, such as lutein and β-carotene, and up to 100 times more than α-tocopherol [1]. ASX could, therefore, play a key role in reducing oxidative stress; oxidative stress is a phenomenon caused by imbalance between production and accumulation of reactive oxygen species (ROS) in cells and tissues and detoxification of these reactive products through specific mechanisms. Oxidative stress is indeed involved in several human pathologies, such as cancer, diabetes, autoimmune, cardiovascular, and neurologic diseases [1,2]. Mechanistically, antioxidant activity may have both enzymatic [3] and non-enzymatic [4,5,6,7] origin, the latter case pertaining, most prominently, to scavenging ROS by chain-breaking antioxidants. The ROS scavenging occurs through diverse scenarios. Among them, the hydrogen atom transfer (HAT) [4,5], single electron transfer-proton transfer (SET-PT) [6], and sequential proton-loss electron transfer (SPLET) [7] mechanisms are noteworthy. HAT is the most favorable antioxidant mechanism in nonpolar environment, while SET-PT and SPLET are the more prevalent mechanisms in polar media [6], including aqueous and hydrophilic surrounding. As the chain-breaking antioxidant, ASX is efficient both under hydrophilic and hydrophobic conditions. It spans the cell-membrane bilayer so that its terminal moieties effectively scavenge ROS on the hydrophilic membrane surface, while its polyene chain traps ROS in the hydrophobic interior of the membrane [8].

The pharmacologic potential of ASX is now well recognised (see Reference [1] for a recent and comprehensive review) and it is not limited to its antioxidant activity. Indeed, ASX can protect animals, human beings included, against a wide range of diseases with excellent safety and tolerability [1]. A number of studies have shown that ASX treatments can modulate the functions of the immune system and control inflammation [1]. Inflammation is a complex immune response initiated by cells of the innate immune system, a group of leukocytes present in all animal tissues, most of which can move freely between tissues and uptake cell debris, foreign particles and invading microorganisms through a process known as phagocytosis [9]. These cells respond to biochemical signals present in the tissue microenvironment by producing a class of proteins known as inflammatory cytokines that, in turn, trigger the inflammatory cascade [10]. Inflammation is an essential physiologic process since it is at the basis of the host defence mechanisms to pathogens and tissue injury. However, excessive uncontrolled inflammation is detrimental to cells and tissues of the host itself where it can cause a pathologic condition which is potentially lethal or at least related to many life-threatening diseases [11].

Inflammation is also involved in tissue fibrosis, a serious long-term side effect of radiation therapy which impairs respiratory, intestinal and urinary functions [12]. Approximately 50% of all cancer patients receives radiotherapy to kill cancer cells and, thus, control their growth and the spread of the disease [13]. Radiation-induced fibrosis (RIF) affects a significant proportion of cancer patients receiving radiotherapy, by some estimates one-quarter of patients [14]. RIF consists in an excessive accumulation of collagen and other extracellular matrix components in the skin, subcutaneous tissues, lung, breast, gastrointestinal and urinary tracts causing multiple symptoms that impact the life quality of the patients [14]. RIF is initiated by direct radiation-induced cell damage and by indirect mechanisms which, in turn, involve the generation of reactive oxygen and nitrogen species (ROS and RNS, respectively) through water radiolysis and activation of nitric oxide synthase [15]. Oxidative stress continues for days or months after the initial exposure to radiation and it does not involve tumour cells only, that are obviously the main target of radiotherapy, but also normal cells in the tissue surrounding the tumour and finally non-targeted bystander cells through intercellular communication mechanisms [16]. Central to RIF is the role of tissue-resident leukocytes, such as macrophages, which, in response to ROS accumulation and tissue damage, activate the expression of pro-inflammatory cytokines, such as transforming growth factor beta (TGFβ) [17]. TGFβ may, in turn, increase ROS production through suppression of antioxidant enzymes, thus leading to a positive feedback that sustains oxidative stress and inflammation [17]. TGFβ, finally, promotes fibroblasts recruitment and local deposition of extracellular matrix components [17].

It has been reported that post-radiation antioxidant therapy significantly reduces RIF in animal models [18]. Because of its acknowledged antioxidant activity, ASX might, therefore, be used to reduce or prevent RIF. ASX, however, is not soluble in water-based biological fluids and is sensitive to temperature, light and oxygen, all factors that limit its stability and bioavailability and that can be controlled by ASX encapsulation into appropriate chemical matrices [19]. Encapsulation of ASX into micrometer-sized particles might also represent an excellent strategy to deliver ASX specifically to macrophages and other innate immune cells that are characterised by their unique ability to engulf, uptake and degrade particles of this size through phagocytosis. To this purpose, we developed protein-based ASX-loaded microparticles, and we explored their biological activity in vitro with macrophage cells derived from a BALB/c mouse, namely J774A.1.

## 2. Results

### 2.1. Characterisation of ASX-Loaded Microparticles

**Figure 1:**
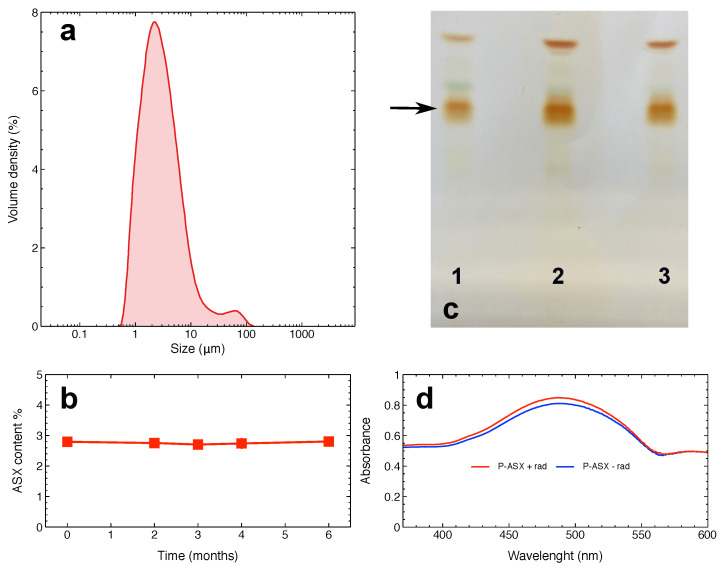
Physical and chemical properties of Astaxanthin (3,3${}^{\prime }$-dihydroxy-β-β${}^{\prime }$-carotene-4,4${}^{\prime }$-dione; ASX)-loaded microparticles. (**a**) Distribution of particles size. (**b**) Storage stability of the particles. This panel shows the ASX oleoresin content in particles stored at −20 °C in the dark under a nitrogen atmosphere for the indicated times. (**c**) Thin layer chromatography of ASX oleoresin (lane 1), of ASX oleoresin extracted from non-irradiated particles (lane 2), or from particles irradiated with a dose of 4 Gy (lane 3). The arrow shows the bands corresponding to mono-esterified ASX above which green-coloured bands corresponding to chlorophyll are visible. The most migrating bands are those of ASX di-esters. (**d**) Absorption spectra of ASX oleoresin extracted from irradiated (red line) or non-irradiated (blue line) particles. In both (**c**),(**d**), differences between irradiated and non-irradiated ASX samples are not appreciable.

ASX oleoresin was encapsulated into particles composed of proteins from whey. Figure 1a shows the size distribution of the particles. The mean and median particles size were 2.8 μm and 2.5 μm, respectively. It is worth noting that specialised cells, such as macrophages, can internalise particles >0.5 μm, with an optimal uptake of particles in the 1–10 μm range [20]. Thus, more than 50% of ASX particles can, in principle, be optimally phagocytised up by J774A.1 macrophages.

ASX microparticles could be safely stored for up to six months under appropriate conditions that protects ASX from heat, light, and oxygen (Figure 1b). In anticipation of future studies involving ionising radiation, the chemical stability of ASX-loaded microparticles were examined following a radiation exposure. Figure 1c,d indicate that encapsulated ASX is not affected by radiation treatments with a dose of 4 Gy as it appears to have the same physico-chemical properties of ASX extracted from non-irradiated particles.

### 2.2. Phagocytosis of ASX-Loaded Microparticles in J774A.1 Macrophages

Oleoresin extracts from *H. pluvialis* are known to emit yellow to red fluorescence when excited by light around 488 nm [21]. This fact can be exploited to measure phagocytosis of ASX-loaded microparticles by fluorescence microscopy and flow cytometry.

**Figure 2:**
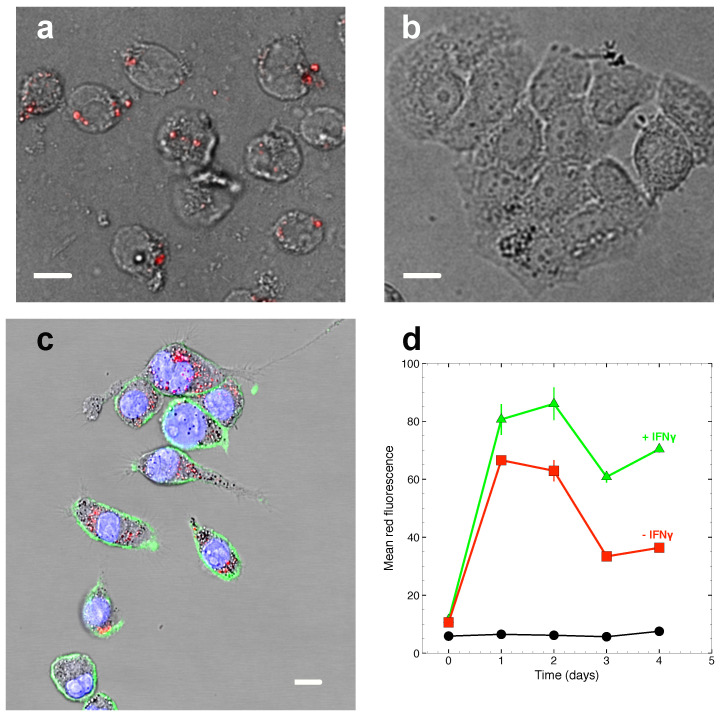
Phagocytosis of ASX-loaded microparticles in J774A.1 macrophages. (**a**),(**b**) Raw fluorescence microscopy images obtained with J774A.1 macrophages (**a**) and T47D tumour cells (**b**). Particles loaded with ASX oleoresin are fluorescent and are found associated with macrophage cells only. (**c**) Mid optical section of J774A.1 cells obtained by confocal microscopy shows intracellular localisation of ASX particles. Green fluorescence: cell membranes labelled with anti-CD11-FITC antibodies; blue fluorescence: cell nuclei; red fluorescence: ASX particles. Fluorescence signals are superimposed to the bright-field optical channel to show cell structures. In (**a**)–(**c**), a white bar 15-μm long has been added to set the microscopic length scale. (**d**) Phagocytosis kinetics as measured by flow cytometry with J774A.1 cells. The data shows that ASX particle fluorescence rapidly accumulates in the cells and that treatment of the cells with INFγ further increases cell associated fluorescence. Black circles: cell autofluorescence; red squares: cells tretated with 56 μg/mL ASX particles at 37 °C; green triangles: cells treated with 56 μg/mL ASX particles and 100 ng/mL INFγ at 37 °C.

Data in Figure 2 show that ASX-loaded microparticles rapidly accumulate in J774A.1 macrophages but not in T47D human breast carcinoma cells that, as all other non-phagocytic cells, cannot internalise compounds of this size. INFγ, a key activator of macrophages, is known to stimulate phagocytosis in myeloid cells [22]. Treatment of J774A.1 cells with INFγ indeed increases cell associated fluorescence and, thus, causes higher accumulation of ASX-loaded microparticles in macrophage cells.

### 2.3. Cytotoxicity of ASX-Loaded Microparticles on J774 A.1 Cells

Empty and ASX-loaded microparticles were added in various doses to cell culture media containing J774A.1 cells. The cells were allowed to grow under the continuous presence of the various treatments and intracellular ATP levels were measured as the function of time.

**Figure 3:**
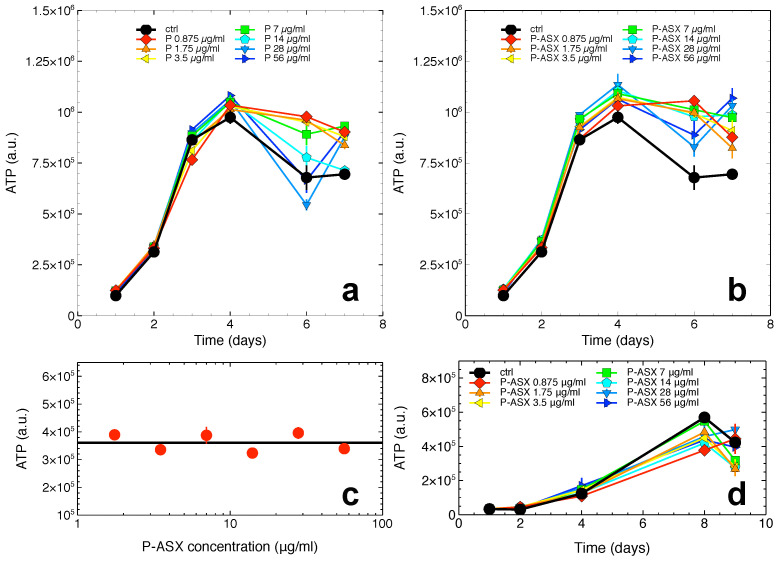
Cytotoxicity of empty and ASX-loaded microparticles. (**a**),(**b**) ATP levels in J774A.1 cells as the function of treatment time with the indicated doses of empty (P, (**a**)) or ASX-loaded (P-ASX, (**b**)) microparticles. In both panels, the growth of untreated control cells is shown for comparison (black line and circles). (**c**) ATP levels for J774A.1 cells treated with ASX-loaded microparticles for 48 h. The particles were previously irradiated with a dose of 4 Gy γ-rays. The black line indicates ATP levels for control untreated cells in this experiment. (**d**) Cytotoxicity of ASX-loaded microparticles as evaluated with T47D tumour cells.

Data in Figure 3 show that both empty and ASX-loaded microparticles were not cytotoxic but rather conferred to cells a slight growth advantage at later times of the J774A.1 cell growth kinetics. This effect was probably due to the whey proteins used to make the particles’ shell that, after phagocytosis and intracellular proteolysis, fed the cells with an extra-source of nutrients. Indeed, this effect was not observed with non-phagocytic T47D cells that do not internalise ASX-loaded microparticles (see, e.g., Figure 2).

In anticipation of future studies involving ASX particles exposed to radiation, the cytotoxicity of irradiated particles was measured. As shown in Figure 3c, the ATP levels in J774A.1 cells exposed to previously irradiated ASX particles were unchanged consistent with irradiated ASX particles being non-toxic to the cells.

Since ASX-loaded microparticles were not cytotoxic in the following experiments we used the particles at the highest assayed dose of 56 μg/mL.

### 2.4. Effects of ASX-Loaded Microparticles on Intracellular ROS Levels

We developed a flow cytometry assay to measure at the single cell level the activity of ASX entrapped in protein particles on intracellular ROS. To modulate intracellular ROS, we treated J774A.1 cells with H${}_{2}$O${}_{2}$. ROS were detected using the fluorescent DCF-DA probe as described in the Section 4.

**Figure 4:**
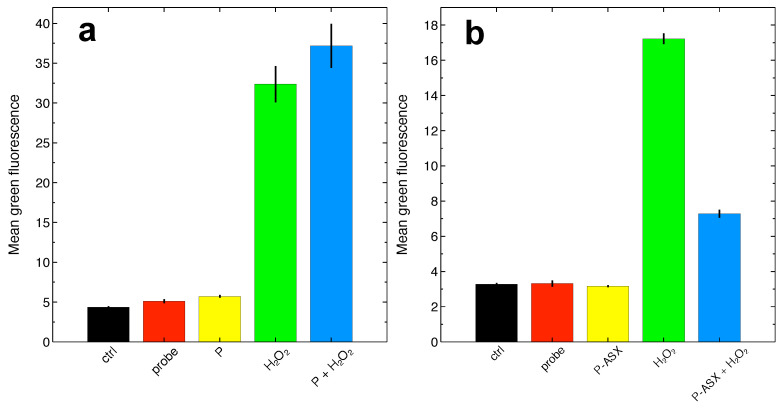
Determination of ROS levels at the single cell level by flow cytometry using the fluorescent probe DCF-DA.(**a**) Experiments carried out with cells treated with empty microparticles (P). Overall, the reported differences are statistically significant ($F_{4,20}=284.5$, $p<2\times 10^{-16}$, ANOVA test). H${}_{2}$O${}_{2}$ significantly increases intracellular ROS with respect to controls, but ROS levels in cells treated with H${}_{2}$O${}_{2}$ and empty particles were not statistically different from cells treated with H${}_{2}$O${}_{2}$ alone, suggesting that, within the power of the measurement, empty particles cannot reverse intracellular H${}_{2}$O${}_{2}$-induced ROS accumulation (p=0.85, Tukey-HSD post-hoc test). (**b**) Experiments carried out with ASX-loaded microparticles (P-ASX). The reported differences are statistically significant ($F_{4,20}=5828$, $p<2\times 10^{-16}$, ANOVA test). ASX-loaded microparticles can significantly reduce intracellular H${}_{2}$O${}_{2}$-induced ROS accumulation ($p<10^{-8}$, Tukey-HSD post-hoc test). In both panels, the labels refer to: ctrl, cell autofluorescence; probe, fluorescence measured with cells loaded with the DCF-DA probe; P or P-ASX, fluorescence measured from cells loaded with DCF-DA and treated with empty (P) or ASX-loaded (P-ASX) microparticles; H${}_{2}$O${}_{2}$, fluorescence measured from cells loaded with DCF-DA and treated with H${}_{2}$O${}_{2}$; P or P-ASX + H${}_{2}$O${}_{2}$, fluorescence measured from cells loaded with DCF-DA, treated with H${}_{2}$O${}_{2}$ and with empty (P) or ASX-loaded (P-ASX) microparticles.

Treatments with ASX-loaded microparticles but not empty particles reduce intracellular ROS accumulation due to H${}_{2}$O${}_{2}$ by ∼75% of control untreated cells (Figure 4). Thus, phagocytosis of ASX particles protects J774A.1 macrophages from oxidative stress.

### 2.5. Effects of Radiation and of ASX-Loaded Microparticles Treatments on J774 A.1 Cells

As shown above, ASX microparticles can protect J774A.1 macrophages from oxidative stress. Intracellular ROS levels also increase as the consequence of water radiolysis in irradiated cells. We, therefore, assayed whether ASX particles could protect J774A.1 from radiation damage. The clonogenic assay is the standard quantitative test in experimental radiation biology [23], but, for their biological characteristics, we were unable to perform this assay with J774A.1 macrophages despite several attempts (see the Section 3 for further discussion on this critical point).

**Figure 5:**
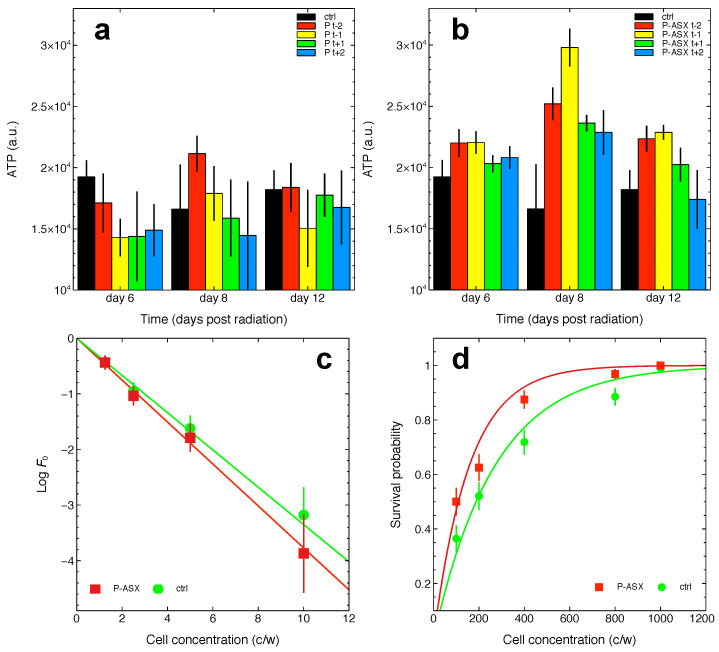
Effects of 56 μg/mL of ASX-loaded microparticles on irradiated J774A.1 cells. (**a**),(**b**) Cells were either left untreated (ctrl) or treated with empty particles (P, (**a**)) or with ASX-loaded microparticles (P-ASX, (**b**)) 2 and 1 days before radiotherapy (−2 and −1, respectively, in both panels) or 1 and 2 days after radiotherapy (+1 and +2, respectively, in both panels) with a dose of 4 Gy γ-rays. ATP was then measured at the indicated time points. (**c**) Limiting dilution assays with untreated cells (ctrl) or with cells treated with ASX microparticles (P-ASX). Linear regression weighted on experimental error of log-transformed data with the linearised model described by Equation (2) allows to estimate the clonogenic potential of the cells (i.e., parameter ϵ in Equation (2)) under both treatment conditions. The results are: ctrl, $\epsilon_{ctrl}=0.335\pm 0.029$, $r^{2}=0.99$; P-ASX, $\epsilon_{P-ASX}=0.377\pm 0.033$, $r^{2}=0.99$. Linear regression of all data factorised per treatment with an extended linear model let us to conclude that the difference between $\epsilon_{ctrl}$ and $\epsilon_{P-ASX}$ is not statistically significant (p=0.148). (**d**) Survival probability of independent populations of untreated (ctrl) or ASX microparticles (P-ASX) treated cells using the method described in the Section 4 and developed in Reference [24]. Nonlinear fits of experimental data with Equation (1) allows to estimate the fraction of the cells surviving radiation treatments (here a 4 Gy dose of γ-rays). The results are: ctrl, $S{\left(D\right)}_{ctrl}=0.0110\pm 0.0007$, goodness-of-fit statistics $\chi^{2}/df=2.05$; P-ASX, $S{\left(D\right)}_{P-ASX}=0.0168\pm 0.001$, goodness-of-fit statistics $\chi^{2}/df=2.9$. The difference between $S{\left(D\right)}_{ctrl}$ and $S{\left(D\right)}_{P-ASX}$ was statistically significant (Z=4.49, $p=3.5\cdot 10^{-6}$).

We first investigated if ASX microparticles could confer to irradiated J774A.1 a selective advantage in long-term growth assays. As shown in Figure 5, treatment with ASX—but not empty microparticles—provides a growth advantage to irradiated macrophages as evaluated by ATP content of the cell populations. The maximum effect is observed for cells treated with ASX microparticles 1 day before radiation treatment (Figure 5). The effect appears to be transient because in these assays cells are cultured in a closed environment, and, at later times, the toxic effects caused by nutrient deprivation and accumulation of waste molecules in growth media dominate cell growth kinetics.

We also quantified the radio-protective effects of ASX particles using a recently-developed experimental approach to measure the survival probability of irradiated cells [24]. The method is based on a probabilistic model of the cell survival after radiotherapy (see the Section 4) and, importantly, it takes into account the self-renewing (i.e., clonogenic) potential of non-irradiated cell clones which must be determined in independent limiting dilution assays. This is an important point in the present case because, as shown in Figure 2, ASX microparticles can positively influence cell survival of non-irradiated cells. This is also confirmed in Figure 5 that shows a slightly higher, albeit not significant, clonogenic potential of cells treated with ASX microparticles (37.7±3.3%) as compared to control untreated cells (33.5±2.9%).

Nonlinear fit of the data with Equation (1) indicates that the surviving fraction S(D) of cells exposed to 4 Gy γ-rays and treated with ASX microparticles is ∼1.53 times higher with respect to that of control cells. Thus, ASX microparticles confer a slight but significant survival advantage to cells irradiated with a dose of 4 Gy γ-rays.

Experiments shown in Figure 5c,d require a careful measurement of the number of cells seeded in each well of multiwell culture plates that are subjected to radiation. Since ASX microparticles affect cell growth (also see Figure 2), particles were added to cells just before radiation treatments. As shown in Figure 5b, this might not be the best choice as ASX microparticles appear more efficient in protecting cells if given one day before radiation.

### 2.6. Secretion of Bioactive TGFβ by Macrophages Treated with ASX-Loaded Microparticles

Secretion of active TGFβ in culture supernatants of J774A.1 cells was evaluated using a bioassay based on MFB-F11 cells [25]. Cells release a protein complex formed by TGFβ and LAP, and this complex must dissociate to allow TGFβ to bind its receptor and start the intracellular signalling cascade in target cells. Dissociation of the protein complex is usually obtained by acid denaturation of the LAP peptide [25]. MFB-F11 cells have been engineered to activate secreted alkaline phosphatase (SEAP) transcription and secretion in response to TGFβ binding to its receptor [25]. Thus, SEAP quantification in the culture supernatants of MFB-F11 cells is proportional to the amount of active TGFβ secreted by J774A.1 cells.

**Figure 6:**
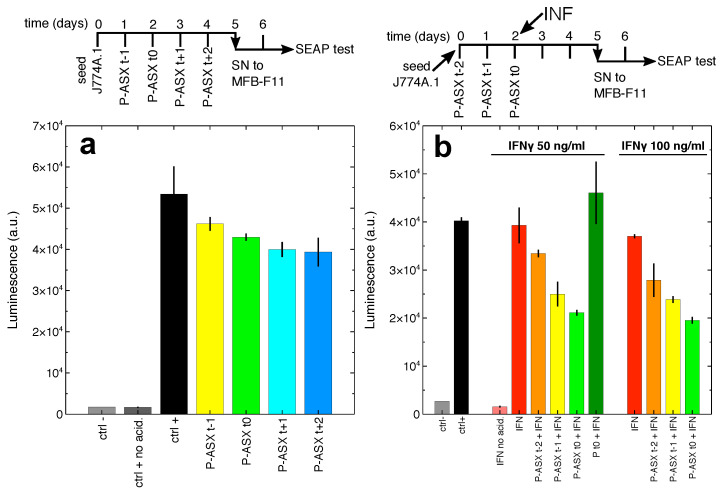
Treatment of J774A.1 macrophages with ASX-loaded microparticles inhibits TGFβ secretion. (**a**) The cells were treated with ASX microparticles following the scheme sketched on top of the panel. At day 5 the supernatants from J774A.1 cells were administered to MBF-F11 cells and after 2 more days secreted alkaline phosphatase (SEAP) activity in the culture supernatants (SN) of these cells was measured. Bar labels refer to: ctrl −, untreated MBF-F11 cells; ctrl +, MBF-F11 cells treated with J774A.1 cells supernatants subjected to acid denaturation (to dissociate the TGFβ-LAP complex and activate the cytokine); ctrl + no acid, in this case J774A.1 cells supernatants are not subjected to acid denaturation; P-ASX (t − 1, t0, t + 1, t + 2), treatment of J774A.1 cells with ASX microparticles at the time points indicated on top of the panel. The differences in the data shown in this panel are statistically significant ($F_{5,11}=41.8$, $p=8.6\times 10^{-7}$, ANOVA test). Only ASX particles given at day 3 and 4 after J774A.1 cell seeding significantly inhibit TGFβ secretion (p=0.027 and p=0.02, respectively, Tukey-HSD post-hoc test). (**b**) The cells were treated with ASX microparticles following a slightly modified scheme with respect to that described above. As shown in the diagram on top of the panel, at day 2 the cells were also treated with two different concentrations of IFNγ (interferon gamma). Inhibition of TGFβ secretion by ASX microparticles is greater than that observed in panel (**a**), an effect that is probably correlated to the higher uptake of the particles in IFNγ-treated macrophages (see, e.g., Figure 2). Bar labels are as in panel (**a**) with the following additions: IFN no acid, MBF-F11 cells treated with supernatants, not subjected to acid denaturation, from IFNγ-stimulated J774A.1 cells; IFN, MBF-F11 cells treated with acid-denatured supernatants from IFNγ-stimulated J774A.1; Pt0+IFN, MBF-F11 cells treated with acid-denatured supernatants from J774A.1 cells treated with IFNγ and empty microparticles. Treatment with IFNγ at 50 ng/mL does not significantly increase bioactive TGFβ secretion by J774A.1 cells but significantly increases the inhibitory effect of ASX microparticles administered at day 1 and 2 after J774A.1 cell seeding (p=0.006 and p=0.001, respectively, Tukey-HSD post-hoc test). Treatment with IFNγ at 100 ng/mL do not significantly increase bioactive TGFβ secretion by J774A.1 cells but significantly increases the inhibitory effect of ASX microparticles administered at day 0, 1, and 2 after J774A.1 cell seeding (p=0.04, p=0.003 and p=0.0006, respectively, Tukey-HSD post-hoc test).

J774A.1 macrophages constitutively secrete TGFβ [26]. Indeed the supernatants of J774A.1 cells, when subjected to acid denaturation, induces SEAP release by MFB-F11 cells, thus revealing TGFβ-LAP complex secretion by J774A.1 cells (Figure 6a).

Treatment of J774A.1 macrophages with ASX-loaded microparticles significantly reduces the accumulation of bioactive TGFβ in culture supernatants. IFNγ (interferon gamma) does not improve the release of TGFβ-LAP complexes from J774A.1 cells (Figure 6b). However, as also shown in Figure 2, IFNγ can increase cellular uptake of ASX-loaded microparticles and, as the consequence, their effect on TGFβ secretion. Indeed, a significantly greater reduction of bioavailable TGFβ is observed when J774A.1 cells are treated with both IFNγ and ASX microparticles (Figure 6b). In addition, a higher concentration of IFNγ anticipates the inhibitory effect of ASX microparticles on bioactive TGFβ secretion. Of note, empty particles do not significantly affect TGFβ secretion either in untreated J774A.1 cells (p=0.29, one-way ANOVA followed by Tukey-HSD post-hoc test) and in cells treated with 50 ng/mL IFNγ (p=0.12, one-way ANOVA followed by Tukey-HSD post-hoc test) as shown in Figure 6b.

**Figure 7:**
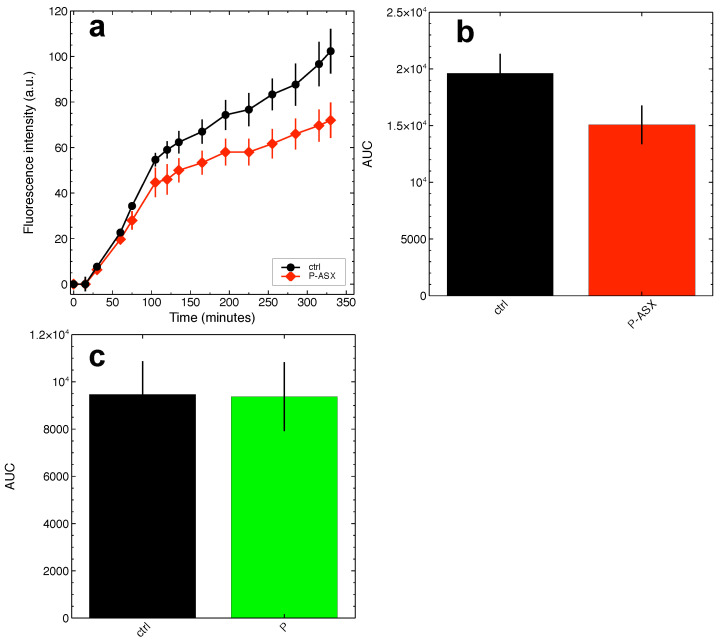
ASX-loaded microparticles inhibit the activity of intracellular convertases in J774A.1 cells. The activity of intracellular convertases was measured in cell extracts using the convertases-specific fluorogenic substrate Boc-RVRR-AMC. (**a**) Fluorescence kinetics measured for cells treated with ASX-loaded microparticles (P-ASX) or left untreated (ctrl). (**b**),(**c**) The overall amount of substrate conversion by convertases was quantified as the area beneath the fluorescence kinetics curves (AUC) as those shown in (**a**) and obtained in independent assays with cells treated with ASX-loaded particles (P-ASX, (**b**)) or empty particles (P, (**c**)). ASX treatments significantly inhibit convertase activity ((**b**), p=0.028 Student-t test), an effect that is not observed for empty particles ((**c**), p=0.94 Student-t test).

TGFβ is synthesised as a single pro-peptide molecule that is then processed along the intracellular secretory pathways by furin convertase, a proteolytic enzyme that cuts the pro-peptide to form a complex made of mature TGFβ and the LAP peptide [27]. Several plant diterpene molecules inhibit furin [28], and it is tempting to speculate that ASX might inhibit it, as well being a tetraterpene molecule. This might explain the findings shown in Figure 6, since unprocessed TGFβ is not bioactive on target cells. According to this molecular hypothesis, J774A.1 macrophages treated with ASX-loaded microparticles show significant inhibition of protein convertase activity as evaluated by time-dependent cleavage of the convertases-specific fluorogenic substrate Boc-RVRR-AMC (Figure 7). The results indicate that the reduction of active TGFβ secretion might indeed be explained by ASX inhibition of intracellular convertases. It is worth noting that the experiments shown in Figure 7 might underestimate the inhibitory activity of ASX (see the Section 3 for further comments on this point).

## 3. Discussion

RIF is a long-term side effect of radiation therapy of cancer [12], and it remains a major obstacle for thoracic radiotherapy of lung, esophageal and breast cancers where it causes serious lung injuries in up to 37% of treated patients [29]. Fibrosis induced by external beam radiation, however, impairs also gastrointestinal and urinary functions and more generally it may manifest as a systemic disorder that involves the skin, muscles, joints, mucosae, lymphatic systems [12]. RIF is a complex multifactorial disorder, but, mechanistically, the cytokine TGFβ appears as a central player [12]. Indeed, it has been shown in vivo that inhibition of TGFβ receptor signalling reduces inflammation and lung fibrosis [12], and clinical trials with specific kinase inhibitors and other anti-inflammatory drugs are presently ongoing [12].

Single therapeutic approaches are unlikely to solve multifactorial disorders, and the severity of the symptoms associated with RIF claims for efforts in developing novel therapeutic strategy that could help to keep under control the side effects of radiotherapy. The present study may contribute to this purpose. When encapsulated into microparticles, ASX can be delivered specifically to phagocytic cells, such as macrophages, to inhibit intracellular ROS levels and production of bioavailable TGFβ. While ROS scavenging by ASX is well characterised, the observed inhibition of TGFβ release remains to be fully explained at the molecular level. We show here that ASX particles can inhibit the activity of intracellular convertases, a class of enzymes that is important for the post-translational processing and activation of numerous pro-proteins [28]. TGFβ is activated by furin convertase along the secretory pathway, and the observed enzyme inhibition by ASX might explain the reduced secretion of active TGFβ by the cells. However, to the best of our knowledge, no probes are available to specifically measure furin activity, and the Boc-RVRR-AMC substrate used here is not an exception. Thus, the present results do not formally provide a demonstration of the molecular events behind the observed ASX effects on TGFβ. ASX inhibition of intracellular convertases might, nonetheless, have been underestimated in our assays. We observed that only tiny amounts of the widely exploited convertases-specific substrate Boc-RVRR-AMC are internalised in J774A.1 cells, and this did not allow us to monitor convertases activity in living cells. The activity of these enzymes could only be measured on cell extracts, and cell lysis might have altered the chemical equilibrium between ASX and convertases, thus masking at least part of the ASX inhibitory activity on these enzymes.

Our data also show that ASX-loaded microparticles are resistant to radiation, are not cytotoxic, and they confer to irradiated cells a limited but significant selective survival advantage. The last point deserves further discussion. As mentioned in the Section 2, clonogenic assays are conventionally used in radiobiology to test radiation effects on target cells in vitro. In these assays, cells are initially seeded at appropriate dilutions to let them grow in isolation. Individual cells proliferate and form colonies, and the effects of radiation are quantified by counting the colonies that survive treatments [23]. Unfortunately, we noted that J774A.1 cells show elevated mobility when they are grown on the plastic surface of culture plates; thus, they do not form colonies at all. We also tried to embed the cells into polymeric matrices in order to reduce their mobility, but, even in these cases, we did not succeed in performing appropriate clonogenic assays. We, therefore, decided to resort to a novel experimental approach that allows to measure the survival probability of irradiated cells with great precision but at the costs of more cumbersome experiments [24].

Phagocytes colonise all tissues and play a key role in physiology and pathology. Microparticles might be delivered to them by inhalation through aerosols, by injection or given topically on skin lesions under the form of cream preparations. Overall, the present study shows that it is possible to encapsulate hydrophobic bioactive substances into micrometer-sized particles to target phagocytic cells and modulate their own functions. In particular, our results suggest that ASX microparticles interfere with the positive feedback between ROS and TGFβ in phagocytic cells and treatment strategies involving ASX microparticles should be further studied to examine their potential to reduce inflammation and inhibit radiation-induced fibrosis.

## 4. Materials and Methods

### 4.1. Astaxanthin and Microparticles

Astaxanthin Oleoresin (ASTAPure^®^ 10% Oeloresin from *Haematococcus pluvialis*) was provided by Algatech (Ketura, Israel).

Astaxanthin-loaded microparticles were obtained by emulsification and solvent evaporation approach described in the PCT/IB2019/059991 application, using whey protein isolate (WPI, Carbery Food Ingredients, Ballineen, Ireland) and maltodextrins MD19 (Agrana Staerke GmbH, Gmünd, Austria) as shell materials. The particles were dried using a Mini-Spray dryer B-290 (Büchi Labortechnik AG, Flawil, Switzerland).

ASX in the microparticles was measured with an Evolution 201 spectrophotometer (Thermo Scientific, Waltham, MA, USA) at a wavelength of 480 nm as described in Reference [19]. For quantification purposes, the particles were digested enzymatically with trypsin (Sigma-Aldrich, Merck KGaA, Darmstadt, Germany) at a final concentration of 2 mg/mL in phosphate buffer pH 7.0 for 4 h at 37 °C and ASX extracted with 2 volumes of ethyl acetate (Sigma-Aldrich) for 60 min.

Particle size was measured on dried samples upon dispersion in distilled water by laser diffractometry using a Malvern Master Sizer 3000 (Malvern Panalytical, Worcestershire, UK) with the following settings: particle refractive index, 1.9; particles absorption index, 0.01; water refractive index, 1.33; laser obscuration 12%.

In this paper, the concentration of ASX-loaded microparticles has been expressed in terms of μg of dried particles/ml of solvent. As also shown in Figure 1b, ASX contributes to ∼2.9% particle mass. Since the molecular mass of ASX is 596.8 g/mol, this corresponds to the concentration ratio of 48.6 nM ASX/(μg/mL) dried particles.

### 4.2. Cells and Cell Culture

J774A.1 (murine macrophage) and T47D (human breast carcinoma) cell lines were obtained from the European Collection of Authenticated Cell Cultures (ECACC, Salisbury, UK; ECACC numbers 91051511 and 85102201, respectively). Cells were cultured in RPMI 1640 medium (Biochrom AG, Berlin, Germany) supplemented with 10% heat-inactivated foetal bovine serum (FBS), 2 mM glutamine (Sima-Aldrich, St. Louis, MI, USA), and 10 mg/mL gentamicin (Biochrom) at 37 °C in a humidified 5% CO${}_{2}$ atmosphere.

The mouse embryonic fibroblast MFB-F11 cell line [25] was kindly provided by Dr. Tony Wyss-Coray (Standford University, Standford, CA, USA). The cells were cultured as described above, but, in culture media, we added 125 μg/mL hygromycin B Gold (InvivoGen, San Diego, CA, USA), as indicated [25].

Cells were routinely tested and shown to be negative for mycoplasma contamination using MycoAlert PLUS Mycoplasma Detection Kit (Lonza Walkersville Inc., Walkersville, MD, USA).

### 4.3. Microscopy

Cell morphology was routinely checked using an Evos (AMG, Life Technology) digital inverted microscope. For confocal microscopy analyses, we grew J774A.1 and T47D cells in glass bottom μ-Slide IbiTreat chambers (Ibidi GmBH, Martinsried, Germany; 10,000 cells/well). Cells were treated with 56 μg/mL ASX-loaded microparticles. After 24 h at 37 °C, the cell samples were washed twice with cold sterile PBS and cell membranes stained with an anti-CD11-FITC monoclonal antibody (clone M1/70, InvitroGen, Carlsbad, CA, USA) for 30 min at 4 °C. Cells were then washed with PBS to remove excess antibody and fixed with 400 μL paraformaldehyde for 10 min at 37 °C. Cells were rinsed 3 times with PBS and permeabilised with 0.1% Triton in 2% PBS-BSA. Nuclei were stained with 1:4000 DAPI (4${}^{\prime }$,6-diamidino-2-phenylindole; Sigma-Aldrich, St. Louis, MI, USA) for 10 min at 37 °C and finally washed with PBS. Images were taken with an SP5 confocal microscope from Leica Microsystems (Mannheim, Germany) equipped with a 63× objective (HCX PL APO λ blue 1.4NA OIL). We collected stacks of images at Δz = 1 μm. Image analyses were performed with ImageJ 1.47v software.

### 4.4. Cytotoxicity Assays

Cells were seeded at 5000 cells/well in flat-bottom 96-wells plates in 180 μL RPMI medium containing serial dilutions of ASX-loaded particles or empty particles. Some cell samples were also left untreated for control. The incubation was prolonged for several days, and at each time point intracellular ATP was measured using the Cell Titer Glo^®^ Luminescent Cell Viability Assay (Promega, Milan, Italy) following the manufacturer’s instruction. Luminescence was measured with an FL${}_{X}$ 800 Microplate reader (FL${}_{X}$800, Bio-Tek Instruments, Bad Friedrichshall, Germany). All measurements were carried out at least in triplicate.

### 4.5. Flow Cytometry

A Guava easyCyte 5 flow cytometer (Merck Millipore, Billerica, MA, USA) was used. The instrument is equipped with a 488 nm, 20 mW, blue laser light. Light scattering is measured by means of a forward scatter (FSC) photodiode and a side scatter (SSC) photomultiplier. Three fluorescence channels, green yellow and red, allow to collect cell-associated fluorescence at the same time, thanks to the following filters: green, 525/30 filter; yellow, 583/26; red, 680/30. Instrument calibration was routinely carried out using the Guava EasyCheck kit (Merck Millipore, Billerica, MA, USA) following the manufacturer’s instructions. Raw listmode data were analysed with the software Mathematica (Wolfram Research Inc., Champaign, IL, USA).

### 4.6. ROS Measurements

Cell-associated ROS levels were measured by flow cytometry using the cell permeable 2${}^{\prime }$,7${}^{\prime }$-Dichlorofluorescein diacetate (DCF-DA) fluorescent probe (Sigma-Aldrich, St. Louis, MO, USA). Hydrolysis of the diacetate group by cellular esterase blocks the probe into the cell, thus allowing to measure intracellular ROS levels [30]. Cells were seeded at 6 × 10${}^{4}$ cells/well into the wells of 6-wells culture plates in 3 mL growth medium and treated with 56 μg/mL ASX-loaded particles or empty particles for 5 h at 37 °C to allow phagocytosis. After washings with PBS cells were incubated with 500 μL of 50 μM DCF-DA for 30 min at 37 °C and further washed two times. Cells were then incubated with 3 mL of a solution containing 2.5 complete medium and 0.5 mL of 0.1% (*w/w*) hydrogen peroxide in PBS (final H${}_{2}$O${}_{2}$ concentration of ∼0.017%) to increase intracellular ROS levels and mimic oxidative stress.

### 4.7. Irradiation

Irradiation of cell samples was performed with a Gammacell40 irradiator (Atomic Energy of Canada Limited, Kanata, ON, Canada) equipped with a ${}^{137}$Cs source. The dose rate was 0.6654 Gy/min and the measured uniformity was ±1.3% over the entire sample chamber. Both parameters are monitored by the Radiation Protection Service of the University of Verona.

Control non-irradiated ASX and cell samples were always kept in the irradiator room for the whole duration of the radiation treatments and processed in parallel with treated samples.

### 4.8. Analysis of Irradiated ASX-Loaded Microparticles by Thin Layer Chromatography

ASX-loaded microparticles were dissolved in RPMI medium and treated with a dose of 4 Gy γ-rays. The samples were then mixed with ethyl acetate (Sigma) at 1:2 volume ratio under stirring for 10 min at room temperature and finally sonicated for 1.5 h in an ultrasonic sonicator bath. The aqueous and the organic phases were then separated by centrifugation at 15,000 rpm for 5 min, the organic phase containing ASX oleoresin collected and ethyl acetate removed by evaporation in an N${}_{2}$ saturated atmosphere.

Thin layer chromatography (TLC) was performed on silica gel F${}_{254}$ TLC aluminium sheets (Merck, Darmstadt, Germany) using as the elution solvent a mixture of hexane (60%) and acetone (40%).

### 4.9. Effects of Radiation on Cells: Statistical Models and Experiments

Quantification of radiation effects on J774A.1 cells was carried out as described earlier using an approach based on a novel probabilistic model [24]. Briefly, the model computes the mean number of clonogens that survive irradiation as S(D)ϵμ, where S(D) is the survival probability of clonogens (i.e., cells with self-renewing potential) irradiated with a dose *D*, and ϵ is the fraction of clonoges in a population of μ cells on average. If the cells are randomly and independently distributed, then the probability that they survive a given dose of radiation follows the Poisson distribution. Therefore, the probability that no clonogen survives is:

$$P_{0}=e^{-S(D)\epsilon \mu },$$

and the probability that at least one clonogen survives is:

(1) $$P=1-P_{0}=1-e^{-S(D)\epsilon \mu }.$$

Equation (1) can be straightforwardly translated into experiments by seeding the cells at different densities μ into a high number of independent wells. After ∼20 days post-irradiation with 4 Gy cell populations were scored as surviving or sterilised by careful microscopic analysis. These were carried out by two independent and blinded observers. The overall survival probability *P* was estimated as the ratio between the number of wells containing alive growing cells and the total number of seeded wells.

The survival probability of the clonogens S(D) was then estimated by nonlinear fitting of Equation (1) to data. Precise estimation of this quantity, however, requires independent measurements of the multiplicative parameter ϵ, i.e., of the number of clonogens in the cell population. This was carried out by limiting dilution assays [31]. The cells were seeded at different concentrations into the wells of 96-wells microplates. We prepared one plate for each tested cell concentration ranging from 1 to 20 cells/well. After 20–30 days, the wells showing absence of proliferating cell populations were counted. If the cells are randomly and indipendently distributed, then the fraction of negative wells (the ratio between the number of negative wells and the total number of seeded wells) obeys Poisson statistics [31]:

(2) $$F_{0}=e^{-\epsilon \mu },$$

where $F_{0}$ is the fraction of negative wells, μ the mean cell concentration expressed as the number of initially seeded cells per well, and ϵ is the fraction of clonogenic cells. The parameter ϵ was estimated by fitting Equation (2) to experimental data.

### 4.10. Measuring Bioactive TGFβ

Bioactive TGFβ was measured with a bioassay based on MFB-F11 cells [25]. These are fibroblasts cells isolated from *Tgfb1*${}^{-/-}$ mouse embryos and stably transfected with a synthetic promoter element containing twelve CAGA boxes fused to a secreted alkaline phosphatase (SEAP) reporter gene. CAGA boxes are directly bound by activated Smad3, which, in turn, is activated through phosphorylation when TGFβ binds its receptor [25]. TGFβ is secreted by cells in association with a Latency Associated Peptide [25] that has to be removed, e.g., by acid denaturation, to allow the binding of the cytokine to its receptor.

J774A.1 cells were seeded into the wells of 96-wells culture plates at a density of 2000 cells/well and treated as described in the Section 2. One hundred μL of culture supernatants were carefully collected, centrifuged to remove cellular debris, and treated with 5 μL of 6M HCl solution at room temperature for 10 min to allow activation of TGFβ. The solutions were neutralised to pH 7.4 with the addition of 6M NaOH [25]. Fifty microliters of activated and neutralised supernatants were then given to MFB-F11 previously seeded at 10,000 cells/well into the wells of flat-bottom 96-wells culture plates in 150 μL growth medium. After 48 h, the supernatants of MFB-F11 cultures were collected, centrifuged to remove any cellular debris, and SEAP activity measured using the chemiluminescent SEAP Reporter Gene Assay kit (Roche Diagnostic GmbH, Mannheim, Germany) following the manufacturer’s instructions.

### 4.11. Convertases Activity Assay

J774A.1 cells were seeded at 10,000 c/w in a 96-well plate. Cells were cultured with and without 56 μg/mL of astaxanthin-loaded microparticles or empty microparticles. After 4 h of incubation, cells were washed with PBS and finally resuspended in assay buffer (complete standard growth medium containing 0.25% Triton X-100, CaCl${}_{2}$ 1 mM). The convertases-specific fluorogenic substrate N-t-butoxycarbonyl-Arg-Val-Arg-Arg-7-amino-4-methylcoumarine (Boc-RVRR-AMC, Enzo Life Sciences, Farmingdale, NY, USA) was added at the final concentration of 100 μM. Fluorescence was recorded for 4 h at 37 °C using the FL${}_{X}$ 800 Microplate reader (excitation wavelength 360 nm; emission wavelength 460 nm). The background fluorescence of the substrate in the absence of cell extracts was measured in parallel and subtracted from the data.

### 4.12. Statistics

All assays were carried out in triplicate and repeated at least three times with different cell batches. Data were expressed as mean ± SE, where SE is the standard error of the mean. Statistical analyses have been performed with the open source software for statistical computing and graphics R (version 3.6.0) run under the free integrated development environment RStudio (version 1.0.153).

Nonlinear regression was carried out using the software Mathematica (v.12, Wolfram Research Inc., Champaign, IL, USA). The reduced $\chi^{2}$, i.e., $\chi^{2}/df$ where df is the number of degrees of freedom, was used to determine the goodness of the nonlinear fits.

## Data Availability

Data are contained within the article.

## Abbreviations

The following abbreviations are used in this manuscript:

RIF	Radiation-induced fibrosis
SN	Culture supernatants
ASX	Astaxanthin
ROS	Reactive oxygen species
RNS	Reactive nitrogen species
TGFβ	Transforming growth factor beta
LAP	Latency associated peptide
IFNγ	Interferon gamma
SEAP	Secreted alkaline phosphatase
FITC	Fluorescein isothiocyanate
DAPI	4${}^{\prime }$,6-Diamidino-2-phenylindole
DCF-DA	2${}^{\prime }$,7${}^{\prime }$-Dichlorofluorescein diacetate
SSC	Side scatter
FSC	Forward angle light-scatter
